# Conferring Honorary Degrees

**Published:** 1902-05

**Authors:** 


### Editorial.

### CONFERRING HONORARY DEGREES.

In the March number of *Items of Interest* the editor publishes a letter from Price Cheaney, M.D., D.D.S., of Dallas, Tex., in which he says, "It is the need of some arrangement by which the large number of old practitioners who have devoted a great part of their lives to the profession of dentistry, but who, through the fault of circumstances, have never been able to graduate." He recognizes the difficulties surrounding this whole question, but he makes the following suggestion: "Let the National Association of Dental Faculties appoint an examining committee from their Association, not having over one member from any one college, to meet at the same time or directly before or after their meeting; and let them examine applicants for advanced standing and give certificates admitting to the second, third, or fourth years, as the circumstances may warrant, and let this certificate be accepted by any school in the Association."

The editor, in replying to this letter, says, "We may pertinently extend the sphere of this discussion to ask why it is that honorary degrees in dentistry have been discountenanced. Great universities are constantly conferring honorary degrees in Arts, Letters, Law, Theology, and Medicine. Why, then, should it be dangerous practice in dentistry?"

The question that Dr. Cheaney propounds is a very old one, and has caused more heart-burnings with the older practitioners and more uncomfortable hours in the older faculties than the majority of the present generation of dentists have any knowledge. From the time of the organization of the Baltimore College of Dental Surgery, in 1839, and for twenty-five years thereafter, nothing much was heard of this question of college degrees. The older men had the control of the dental organizations, and, with the exception of the few with the medical and dental degrees, they had all received their dental education in the laboratory. The colleges were but few, and the number of graduates was so small that they had failed to produce a marked impression upon associations or upon dental literature. Then the handwriting on the wall came as a prophecy to these men, and, translated, it meant to them, Laws will be passed in all the States of the Union, and this will mean an increase of the numbers of the student body, and in a few short years your influence will have departed. At once began a demand upon the then existing dental colleges for honorary degrees, and this increasingly grew as years were added to years.

It was recognized by some of the schools that dentistry occupied an anomalous position as compared with the older professions. They had centuries behind them. The schools of theology, law, and medicine were hoary with age, but dentistry had but a quarter of a century of dental college training. It was not a profession, and had scarcely reached the degree of an art. It was, therefore, regarded as important that the scattered elements should be brought together and fused, as it were, into a homogeneous body. It was well understood that the majority of men practising dentistry began with the laboratory as the basis of training. Colleges were not, neither were there any laws prohibiting a man from opening an office and practising to the extent of his knowledge. Some were not satisfied with their limited acquirements, and aimed to improve in various ways, but the sporadic nature of these efforts availed but little to elevate the general mass. Then came the period when the increasing number of graduates roused antagonism not only unpleasant individually, but which threatened to be a serious set-back to college training.

The momentous character of the question at issue forced the faculties to consider the advisability of perfecting some plan by which the antagonistic elements could be made to feel that they were a portion of the dental profession. The educational part of dentistry occupied a peculiar position. Some action was required to harmonize matters, but what that should be remained an unsolved problem. The conferring of honorary degrees by wholesale could not be thought of in this connection. It was opening up a traffic in diplomas, or, if confined to the few distinguished, it meant the charge of favoritism. Some schools took this course and many received this degree; others refused.

The Pennsylvania College of Dental Surgery, with which the writer was connected, adopted a different method. The faculty carefully and anxiously considered the question in its relation to the profession. It was finally decided to make examinations upon all the subjects of the curriculum as the basis upon which diplomas were to be granted. When this was made, an open notice was given in the dental periodicals that those having had fifteen years of practice were entitled to come up for an examination, and, passing this, the degree of Doctor of Dental Surgery would be conferred. A considerable number availed themselves of this privilege. It was not supposed that these men could pass the then students' examination, but it was presumed that they could show a general knowledge in the foundation studies. The disappointment was, therefore, great when it was found that, with a few exceptions, the knowledge possessed was confined entirely to practical subjects. This settled the matter, and all attempts to confer the degree upon this class were abandoned.

From this period began a bitter war upon the colleges. There was at this time a large majority of the practitioners in this outside class, and these were naturally antagonistic. They could not enter the schools and spend two or three years, and they could not matriculate upon advanced standing. Thus the matter stood when the Association of Dental Faculties was organized in 1884.

That part of Dr. Cheaney's article in which he advocates an examination is answered by the historical facts as related. It would be far worse to-day were such an examination attempted. Even the graduates of twenty years ago could not pass the examinations given to students at the present period, and when the course is increased to four years their case will be hopeless for all future time.

The Association of Faculties early considered this question, as some of the colleges then desired the power given them to confer the degree upon several men distinguished in dentistry. It was recognized by the Association that the honorary degree was but one remove from the sale for money of diplomas, and the applications were negatived; but notwithstanding this antagonistic reception these applications were repeated, and in one instance conferred without consent of the organization. The question was finally taken up and settled by the adoption of the following rule: "No college connected with this Association shall confer any degree as honorary which is usually granted in due course of study and examination. All former rules on this subject are hereby repealed." It is, therefore, hopeless to expect that this body will ever open the door to any degree, except it be honestly earned, and if it at any time should so far forget its duty to the profession as to repeal this law, the National Association of Dental Examiners, without doubt, would have something to say of a not very agreeable character. From present indications there is no prospect of this being effected, nor is there any pressure in this direction. The men of the past are gradually being replaced by graduates, and it cannot be long before all those who, without degrees, made the splendid fight for the profession of dentistry upon practical lines will have passed from works to a higher reward. When the time comes that all are in practice with the recognized diploma, may it not be a reasonable hope that then the commercial habit of thought, engrafted upon dentistry during this developing period, may be changed to higher conceptions and the true standard of professional life be established? It is from this want of high moral purpose that the dental profession suffers principally to-day. The trade journal naturally looks upon all things from one standard,—trade competition. The man without a diploma stands no chance in practice with the man with one. We may regret the unfortunate condition of this man, but he belongs to the age of beginnings. It is an honorable period, and no one venerates the man of the past more than the writer, but he recognizes the fact that evolution is iconoclastic. The images fondly cherished are broken on the wheel of progress, and from the fragments are developed the new thoughts, the more perfected race, and the more worthy profession. To accomplish this dentistry must never return to the honorary degree. It is the badge of incomplete education.

The editor of *Items of Interest* is mistaken in supposing that the higher medical schools grant honorary degrees. So far as the writer is aware, professional degrees are no longer conferred. The time will surely come when the self-respecting man will decline to receive any honorary title, come from what source it may. The whole question of granting honorary degrees by our great universities requires re-examination; but whether this be done or not, there should be no backward steps made from the advanced position of the Association of Faculties upon this question. Dentistry is too great, too noble a profession to be cheapened by any short road to its highest honors.

